# Small airway dysfunction measured by impulse oscillometry is associated with exacerbations and poor symptom control in patients with asthma treated in a tertiary hospital subspecialist airways disease clinic

**DOI:** 10.3389/falgy.2024.1403894

**Published:** 2024-08-15

**Authors:** Dylan Beinart, Emily S. Y. Goh, Glen Boardman, Li Ping Chung

**Affiliations:** Department of Respiratory Medicine, Fiona Stanley Hospital, Perth, WA, Australia

**Keywords:** asthma, asthma control test, asthma exacerbation, cutoff, impulse oscillometry, lung function test, small airway dysfunction

## Abstract

**Introduction:**

Small airways dysfunction contributes to asthma pathophysiology and clinical outcomes including exacerbations and asthma control. Respiratory oscillometry is a simple, non-invasive and effort independent lung function test that provides vital information about small airway function. However, interpretation and clinical utility of respiratory oscillometry has been in part limited by lack of agreed parameters and the respective cutoffs. The aim of this study was to determine the prevalence of small airways dysfunction based on published impulse oscillometry (IOS) definition in patients with asthma referred to a tertiary asthma clinic and the extent to which it correlates with asthma clinical outcomes.

**Methods:**

We retrospectively reviewed the medical records of all patients with asthma managed in the severe asthma clinic between January 2019 and December 2022 who underwent routine lung function tests with oscillometry and spirometry. Small airways dysfunction was determined from various published IOS parameter cutoffs, and the data were analysed to determine correlations between IOS parameters and asthma outcomes.

**Results:**

Amongst the 148 patients, the prevalence of small airways dysfunction ranged from 53% to 78% depending on the defining oscillometry parameter. All oscillometry parameters correlated with the severity of airflow obstruction (FEV_1_% predicted, *p* < 0.001). Several oscillometry parameters correlated with asthma symptom burden, the strongest correlation was seen for frequency dependent resistance (R_5_–R_20_) with scores of Asthma Control Questionnaire (ACQ6) (Spearman's rank coefficient 0.213, *p* = 0.028) and Asthma Control Test (ACT) (Spearman's rank coefficient −0.248, *p* = 0.012). R_5_–R_20_ was predictive of poor asthma control defined by ACQ6 >1.5 (OR 2.97, *p* = 0.022) or ACT <20 (OR 2.44, *p* = 0.055). Small airways dysfunction defined by R_5_–R_20_ and area under the reactance curve (AX) also significantly increases asthma exacerbation risk (OR 2.60, *p* = 0.02 and OR 2.31, *p* = 0.03 respectively).

**Conclusion:**

Respiratory oscillometry is a sensitive measure of small airways dysfunction that should complement spirometry in the routine assessment of asthma. Small airways dysfunction is highly prevalent in patients with asthma referred to a tertiary asthma clinic. R_5_–R_20_ was the metric most predictive in identifying patients at risk of asthma exacerbations and poor asthma control.

## Introduction

Asthma is a common, chronic respiratory condition characterized by airway inflammation and variable expiratory airflow limitation (1). Extensive study of asthma pathophysiology has disrupted our previous assumptions of a single disease phenotype and revealed asthma's intricate and heterogenous nature (2). Even with these incredible advances, many patients with asthma still experience burdensome symptoms and poor asthma control, despite optimal doses of inhaled corticosteroids (ICS) (3). It is increasingly clear that the peripheral, or “small” airways significantly contribute to these persistent symptoms (4).

The small airways are generally defined as those with a diameter of less than 2 mm and comprise the conducting airways beyond generation 8 as well as airway generations 17–23 in the respiratory acinar zone (5). Small airways are the major site of airway inflammation in asthma and represent the foremost site of airflow limitation in both asthma and chronic obstructive pulmonary disease (COPD) (6, 7). Dysfunction of the small airways has been associated with particular asthma phenotypes and endotypes such as exercise induced symptoms, obesity and night time awakenings (8). Small airway dysfunction is also associated with dyspnea, poor asthma control, exacerbations, and requirements for higher ICS dosing (8, 9). The development of extra-fine particle inhaler therapy has renewed interest in identifying and treating small airways dysfunction. These devices generate particles smaller than 2 µm that result in superior peripheral airway deposition that is less effected by the severity of airflow obstruction and rapid variation in inspiratory airflow (5). A meta-analysis by Sonnappa et al. demonstrated that patients receiving extra-fine particle ICS therapy had a higher chance of achieving asthma control than those patients treated with standard fine-particle ICS therapy. In addition, exacerbation rates were lower amongst patients treated with extra-fine particle ICS and these outcomes were achieved with significantly lower ICS doses (10).

Small airways dysfunction has long been overlooked in routine asthma management because historically it has been difficult to measure and lacks consensus in interpretation of normality compared with disease states. For decades, spirometry with forced expiration has been the mainstay of asthma investigation but has very poor sensitivity and discrimination for small airways dysfunction. It is estimated that 75% of small airways need to be obstructed before changes are evident on spirometry (11). Specialised testing methods such as multiple breath nitrogen washout and alveolar fraction of nitric oxide can provide useful information but are costly, time consuming and difficult to perform. The lack of convenient and sensitive investigations and the subsequent neglect of small airway involvement in asthma management has led some clinicians to label small airways as the “silent zone.”

Respiratory oscillometry including impulse oscillometry (IOS) and forced oscillation technique (FOT) is gradually gaining traction in the management of airways disease and in particular asthma. Impulse oscillometry is a simple, non-invasive and effort independent technique that uses square-wave impulse pressure variations to measure mechanical properties of the airways and lung parenchyma. Certain respiratory oscillometry parameters, such as frequency dependent resistance (R_5_–R_20_), reactance at 5 Hertz (X_5_) and area under the reactance curve (AX) can provide vital information about small airway function, whilst other parameters are key indicators of treatment response and asthma outcomes (12–15). For example, changes in oscillometry measures of resistance and reactance (R_rs_ and X_rs_) have been demonstrated in response to montelukast, ICS and ICS/long-acting beta_2_-agonists (LABA) (16). Changes in oscillometry (specifically R_5_ and X_5_) also correlate with symptoms in patients with poorly controlled asthma receiving ICS/LABA (17). Compared to spirometry, respiratory oscillometry is comparable in predicting oral corticosteroid (OCS) and short-acting beta_2_-agonist (SABA) use, but IOS measures [R_5_–R_20_, AX and resonant frequency (F_res_)] show better correlation with symptom control in patients with moderate to severe asthma (12). Respiratory oscillometry can detect subtle changes in airway function before they are evident on spirometry and may detect peripheral airway dysfunction in patients with normal spirometry (18, 19). Recent data from Sugawara et al. suggests that respiratory oscillometry can stratify patients to small vs. large airway phenotypes and predict superior response to extra-fine particle size inhaled therapy (13, 14).

Despite respiratory oscillometry being a sensitive, easy to perform lung function test, routine use in clinical practice is limited especially amongst adults with asthma. Barriers to routine use in clinical practice include a lack of standardization between devices and difficulty in its interpretation, due to evidence gaps associated with normative values and cutoffs or minimal clinically important differences (16, 20, 21). This is further complicated by the use of multiple ways to describe the change in respiratory oscillometry parameters in clinical studies that have investigated the relationship between respiratory oscillometry and asthma outcomes. Studies have reported changes using absolute values, *z*-scores and percentage change, and at this point in time these are not readily transposable.

To start addressing some of these barriers, we conducted a retrospective review of all patients with asthma referred to a tertiary severe airways clinic who underwent respiratory oscillometry testing. The primary objective of this study was to determine the prevalence of small airways dysfunction in patients with asthma based on routine lung function testing which includes spirometry measures such as the ratio of the forced expiratory volume in the first one second to the forced vital capacity of the lungs (FEV_1_/FVC) and respiratory oscillometry parameters (R_5_–R_20_. AX, X_5_). The secondary objectives of the study were to determine if small airways dysfunction as defined by published IOS cutoffs defining normality correlated with symptom burden and/or asthma exacerbations.

## Methods

This was a single centre, retrospective study of patients referred to a tertiary respiratory clinic, the Severe Airways Disease clinic at the Fiona Stanley Hospital, Western Australia, who completed standard lung function testing as part of their routine outpatient assessments, between January 2019 and December 2022. The study was approved by the Human Research Ethics Committee (HREC) and Research Governance Unit of Fiona Stanley Hospital (RGS5611).

Eligible patients had respiratory specialist confirmed diagnosis of asthma based on compatible symptom history such as wheeze, dyspnea, chest tightness and/or cough in presence of variable expiratory airflow limitation (1, 22), and had completed spirometry and oscillometry, namely IOS, as part of the standard lung function testing. There were no formal exclusion criteria other than the patient needed to have at least one IOS test result conducted at the asthma clinic documented in their medical records.

The medical records of all eligible patients were reviewed, and relevant information was extracted for data analysis. Information collected about each patient included, patient's age, gender, ethnicity, body mass index (BMI), smoking status, asthma symptom scores (e.g., Asthma Control Questionnaire (ACQ6) or Asthma Control Test (ACT)), frequency of asthma exacerbations, asthma medications, severity of asthma (based on GINA criteria) and lung function test results. The ACQ6 version used in this study is the version calculated using 5 symptom items and frequency of rescue bronchodilator use (23).

The primary time point (sentinel date) for information to be extracted about each patient was the first date that the patient had oscillometry performed at this tertiary asthma clinic. In most cases, the lung function tests were done on the same day as respiratory specialist review or within 48 hours prior. Hence, demographic data, lung function test results, asthma symptom scores, medication use etc. were primarily based on the medical records at the sentinel date. Asthma exacerbations were defined as any worsening in asthma symptoms that resulted in the patient being treated with prednisolone and/or antibiotics or resulted in an unscheduled visit to hospital, accident & emergency, or a general practitioner (1, 22). All asthma exacerbations that occurred in the 12 months before and after the sentinel date were recorded and included in the analysis. Lung function test results that were extracted were as follows; pre-bronchodilator spirometry FEV_1_, FEV_1_% predicted, FVC, FVC % predicted, FEV_1_/FVC ratio and Forced Expiratory Flow over the middle half of the FVC (FEF25%–75% predicted); fractional concentration of exhaled nitric oxide (FeNO); and pre-bronchodilator IOS R_5_, R_5_% predicted, R_20_, R_20_% predicted, R_5_–R_20_, percentage change in R_5_–R_20_ (ΔR_5_–R_20_%), AX, respiratory rate (BF), F_res_, X_5_ and X_5_% predicted.

IOS measurements were collected using the impulse oscillometry device Masterscreen IOS, Jaeger, Germany in accordance with the manufacturer's recommendations. Spirometry was performed after IOS testing using Masterscreen PFT Jaeger Germany. The prevalence of small airways dysfunction was based on the following published cutoffs in IOS parameters; R_5_–R_20 _> 0.07 kPa/(L/s), ΔR_5_–R_20_% > 20%, X_5_ < −0.12 kPa/(L/s), X_5_ <Lower Limit of Normal (LLN), F_res_ > 14.2 Hz and AX > 0.44 kPa/L (8, 24–26).

### Statistical analysis

For the primary objective, comparisons of the prevalence of small airways dysfunction in asthma were analysed using Fisher's exact or Pearson's chi-squared test. For the secondary outcomes, continuous data correlations were compared using Spearman's rank coefficient and continuous non-normally distributed data were compared using Mann Whitney-*U* or ANOVA (Kruskal Wallis). As this was a retrospective study that included all eligible patients, there were no power calculations performed. Statistical analysis was performed using Jamovi version 2.2.5.

If the patient did not have relevant data documented in their medical records, for example they did not have an ACT or ACQ6 score documented, they were excluded from that analysis. Similarly, patients were only included in the exacerbation analysis if their medical records documented that either they had an exacerbation in the specified 24 months around the sentinel date or explicitly stated that the patient did not have an asthma exacerbation. That is, the absence of information about exacerbation history was not assumed to equate to no exacerbation having occurred, and these patients were excluded from this analysis.

## Results

One hundred and forty-eight patients with asthma were included in this retrospective study. The majority of patients were female and of Caucasian ethnicity, see Table 1 for demographic and clinical characteristics of the study population. As expected for patients referred to a tertiary asthma clinic, there was a high proportion of patients with severe asthma with 69% of patients being classified as having severe asthma based on GINA criteria (1). Over half, (54%) of patients had at least one documented moderate to severe asthma exacerbation in the 12 months before or after the sentinel date. In terms of maintenance therapies to manage asthma, the majority of patients were being treated with ICS-based combination therapy with equal proportions (44.6%) being treated with ICS/LABA or ICS/LABA/LAMA (long-acting muscarinic antagonist). Of interest, only 15% of patients taking triple therapy were using a single inhaler triple therapy, consistent with guideline recommendations (22).

**Table 1:** Patient demographics, medication use, lung function and prevalence of small airways dysfunction.

Demographics (*N* = 148)	*N* (%)
	^a^Median (IQR)
Age (years)^a^	49.33 (34.81–62.19)
Male/female (%)	55/93 (37.2%/62.8%)
Ethnicity—Caucasian/Other (%)	121/27 (81.8%/18.2%)
BMI (kg/m^2^)^a^	30.09 (24.77–35.36)
Smoking status (%)	
Current	7 (4.7%)
Former	56 (37.8%)
Never	85 (57.4%)
Exacerbation in the preceding or proceeding 12 months (%)	
Yes	80 (54.1%)
No	56 (37.8%)
Not documented (unknown)	12 (8.1%)
Inhaled therapy	*N* (%)
None	8 (5.4%)
ICS monotherapy	7 (4.7%)
ICS/LABA	66 (44.6%)
LAMA/LABA (no ICS)	1 (0.7%)
Single inhaler triple therapy (ICS/LABA/LAMA)	10 (6.8%)
Triple therapy (ICS/LABA/LAMA) using multiple inhalers	66 (44.6%)
Systemic therapy	*N* (%)
Montelukast	28 (18.9%)
Oral corticosteroids (maintenance)	21 (14.2%)
Biologic (monoclonal antibody)	25 (16.9%)
Pulmonary function tests	Median (IQR)
FEV_1_% predicted (%)	73.3 (57.1–85.0)
FVC % predicted (%)	92.9 (78.5–100.8)
FEV_1_/FVC ratio (%)	62.5 (57.1–85.0)
R_5Hz,_ [kPa/(L/s)]	0.54 (0.40–0.75)
R_5Hz_ % predicted (%)	173.15 (133.98–229.38)
R_20Hz_ [kPa/(L/s)]	0.38 (0.30–0.46)
R_20Hz_ % predicted (%)	136.40 (117.08–166.93)
R_5_-R_20_ [kPa/(L/s)]	0.13 (0.08–0.28)
ΔR_5_–R_20_% (%)	35.26 (22.99–59.68)
AX (kPa/L)	0.66 (0.27–2.10)
BF (L/min)	12.98 (10.68–16.49)
F_res_ (Hz)	15.00 (10.75–20.92)
X_5_ [kPa/(L/s)]	−0.17 (−0.28 to −0.11)
X_5_% predicted (%)	177.00 (115.45–248.20)
FeNO (ppb) (*N* = 144)	31.5 (20.8–60.0)
Prevalence of small airway dysfunction	*N* (%)
R_5_–R_20 _> 0.07 kPa/(L/s)	112 (75.7%)
ΔR_5_–R_20_% > 20%	116 (78.4%)
X_5_ < −0.12 kPa/(L/s)	99 (66.9%)
X_5_ < LLN	78 (52.7%)
F_res_ > 14.2 Hz	81 (54.7%
AX > 0.44 kPa/L	96 (64.9%)

Pre-bronchodilator lung function test and oscillometry results are summarised in Table 1. Sixty-four percent of patients had a FEV_1_/FVC ratio <0.70 indicating that the majority of the patient cohort exhibited significant airway obstruction. The prevalence of small airways dysfunction as assessed by oscillometry ranged from 52.7% to 78.4%, with the R_5_–R_20_ and ΔR_5_–R_20_% identifying the highest prevalences. In addition, 72 patients had FEF25%–75% predicted documented, with 74% of these patients having a FEF25%–75% predicted <60%, which is a spirometry-based indicator of small airways dysfunction (27).

**Figure 1:**
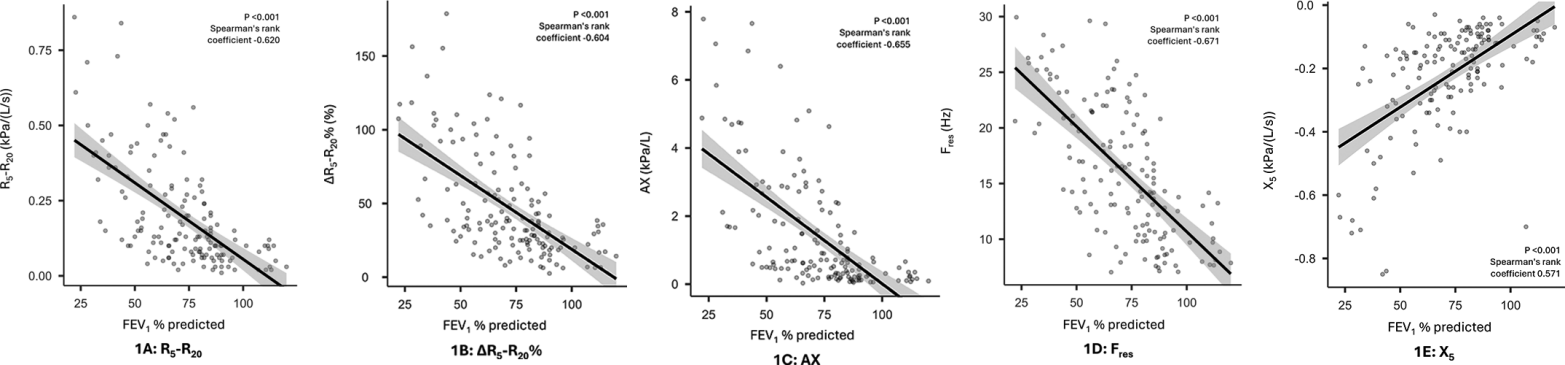
Correlation between IOS markers of small airways dysfunction and airflow obstruction (FEV_1_% predicted) panel 1 (**A**): R_5_–R_20_, 1 (**B**): ΔR_5_–R_20_%, 1 (**C**): AX, 1 (**D)**: F_res_, 1 (**E**): X_5_.

**Figure 2:**
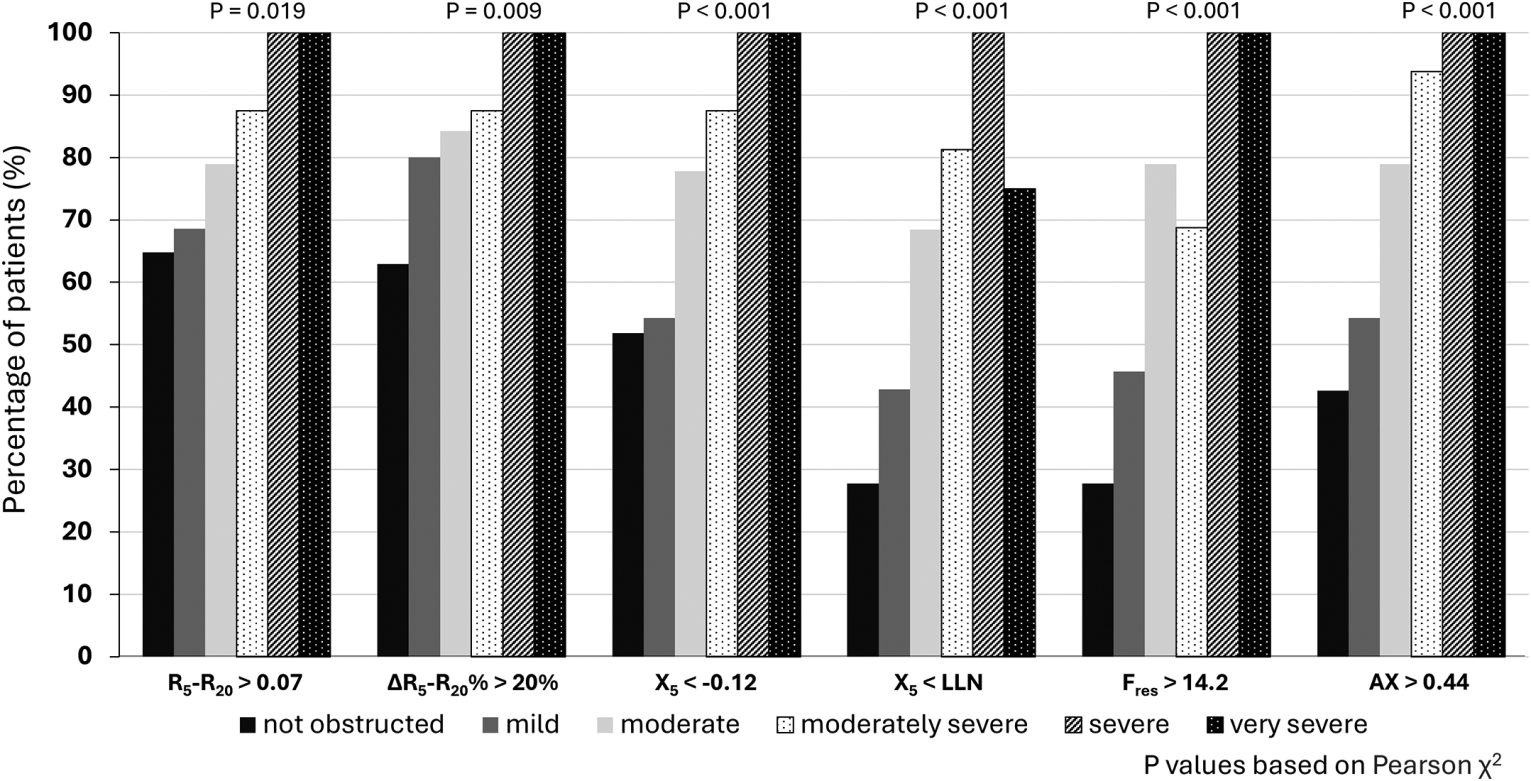
Proportion of patients with IOS defined small airways dysfunction based on severity of airflow obstruction.

In terms of the secondary objectives, small airways dysfunction was correlated with the severity of airflow obstruction. Figure 1 illustrates the inverse relationship between R_5_–R_20_ and FEV_1_% predicted, with the higher R_5_–R_20,_ indicative of more severe small airways dysfunction, being correlated with lower FEV_1_% predicted, indicative of more severe airflow obstruction. Significant correlations (*p* < 0.001) were also observed for other oscillometry markers of small airways dysfunction, see Figure 1. Similarly, while small airway dysfunction was identified in a 27.8%–64.8% of patients with normal spirometry, the prevalence increased with the degree of airflow obstruction (28), see Figure 2.

Small airways dysfunction also correlated with history of asthma exacerbations, with the strongest correlation demonstrated for R_5_–R_20_ (*p* = 0.004), see Table 2. Small airways dysfunction as identified by R_5_–R_20_ and AX more than doubled the odds ratio of a moderate to severe asthma exacerbation occurring within the preceding or proceeding 12 months (Odds Ratio 2.60, *p* = 0.01 and Odds Ratio 2.31, *p* = 0.03 respectively). Other oscillometry indicators of small airways dysfunction, except X_5_ (*p* = 0.102) also demonstrated significant correlations with asthma exacerbations, see Table 2.

**Table 2:** Correlation between small airways dysfunction and asthma exacerbations.

IOS parameter	Exacerbation (*N* = 80) Median (interquartile range)	No-exacerbation (*N* = 56) Median (interquartile range)	*P*-value (Mann Whitney-*U*)
R_5_–R_20_	0.16 (0.09–0.37)	0.12 (0.06–0.19)	0.004
ΔR_5_–R_20_%	38.01 (25.48–82.6)	33.56 (19.69–51.41)	0.014
X_5_	−0.18 (−0.31 to −0.13)	−0.16 (−0.25 to −0.09)	0.102
F_res_	16.65 (12.79–21.57)	13.95 (10.01–18.96)	0.007
AX	0.9 (0.38–2.76)	0.58 (0.21–1.35)	0.010

**Figure 3:**
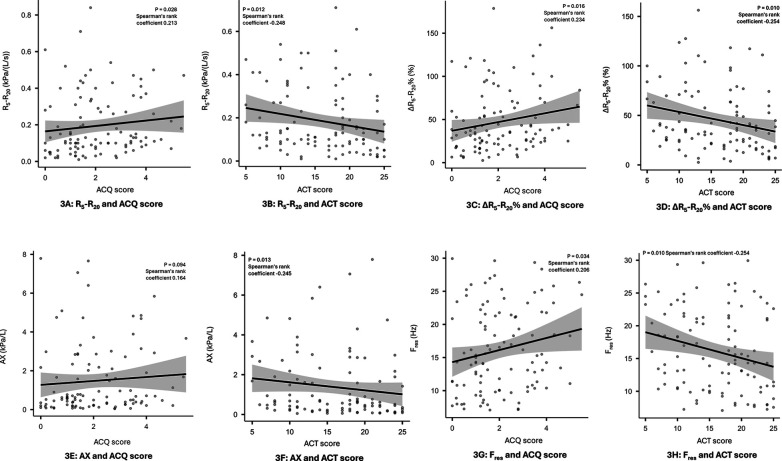
Correlation between small airways dysfunction and symptom burden. Panel 3 (**A**): R_5_–R_20_ and ACQ score, 3 (**B**): R_5_–R_20_ and ACT score, 3 (**C**): ΔR_5_–R_20_% and ACQ score, 3 (**D**): ∆R_5_–R_20_% and ACT score, 3 (**E**): AX and ACQ score, 3 (**F**): AX and ACT score, 3 (**G**): F_res_, and ACQ score, 3 (**H**): F_res_, and ACT score.

Small airways dysfunction was associated with increased symptom burden as assessed by both ACQ6 and ACT scores. Statistically significant correlations were observed for R_5_–R_20_, ΔR_5_–R_20_% and F_res_ with both tests of asthma control, (see Figure 3) while the correlation with AX was only significant for ACT scores and X_5_ failed to demonstrate a significant correlation with either test. Patients with uncontrolled asthma as defined by an ACQ6 score >1.5 were more likely to have small airways dysfunction as identified by R_5_–R_20_ >0.07 and ΔR_5_–R_20_% >20% (Odds Ratio = 2.97, 95% CI 1.19–7.41, *p* = 0.022 and Odds Ratio = 2.69, 95% CI 1.07–6.75, *p* = 0.0039, respectively). Similarly, frequency dependent resistance (R_5_–R_20_ and ΔR_5_–R_20_%) are also predictive of poor asthma control defined by ACT <20 (Odds Ratio 2.44, 95% CI 0.97–6.16, *p* = 0.055 and Odds Ratio 3.36, 95% CI 1.31–8.61 *p* = 0.009, respectively).

## Discussion

The findings of this single-centre, retrospective study of patients with asthma referred to a tertiary severe asthma clinic indicate that small airways dysfunction is highly prevalent amongst this patient cohort. This study adds strength to the body of evidence on the impact of small airways dysfunction on asthma outcomes. Oscillometry tests have predominantly been used in adult population in research settings and abnormality of oscillometry parameters are variably reported as absolute values, *z*-scores or percentage predicted. This study demonstrates that the addition of oscillometry to routine lung function assessment is feasible and contributes to comprehensive asthma assessment in a tertiary hospital setting. Its utility is not limited to diagnosis of small airways dysfunction but shows that frequency dependent resistance [R_5_–R_20 _> 0.07 kPa/(L/s) and ΔR_5_–R_20_% > 20%] also effectively predicts poor asthma symptom control and exacerbation risk. These findings argue for the clinical need for respiratory specialists to incorporate impulse oscillometry into routine clinical practice as it provides additional information about the underlying pathophysiology potentially contributing to the patient's asthma outcomes and choice of more effective treatments.

The prevalence of small airways dysfunction as identified by respiratory oscillometry ranged from 52.7% to 78.4%, and this is higher than that reported in studies where patients were recruited from primary care setting with the prevalences ranging from 30.6% to 42.4% in the Assessment of Small Air-ways Involved in Asthma (ATLANTIS) study (27) and 32.0%–57.4% in a smaller study by Li et al. (29) The higher prevalence of small airways dysfunction in patients referred to respiratory specialists is expected, as this patient cohort is skewed towards patients with severe or difficult to control asthma. It was previously demonstrated that patients with severe asthma defined using British Thoracic Society or the American Thoracic Society/European Respiratory Society criteria have much greater frequency dependent resistance compared with those with milder asthma (30, 31).

In our study the prevalence of small airways dysfunction, as identified by respiratory oscillometry using IOS parameters increased with the severity of airflow obstruction. This finding is consistent with a study conducted by Liang et al, where equivalent inverse relationship between R_5_–R_20_ and FEV_1_ was demonstrated (21). It is also important to recognise that abnormality in small airways was identified via oscillometry even amongst patients with no or mild airflow limitation based on spirometry. Small airways dysfunction is also more prevalent in patients with persistent airflow limitation which negatively impacts on patient outcomes (32). This finding is clinically relevant, as small airways dysfunction is thought to commence early in the clinical course and may not be detected with spirometry until 75% of the small airways are involved (33). Therefore, it may potentially represent a window of opportunity for early intervention before airflow obstruction becomes established.

In this retrospective study, small airways dysfunction as identified by oscillometry was associated with a significant increase in the risk of moderate to severe asthma exacerbations, with abnormalities identified by R_5_–R_20,_ and ΔR_5_–R_20_% more than doubling the odds of an exacerbation. Despite differences in oscillometry devices and quantitative reporting of the parameters, our finding is consistent with the longitudinal data from the ATLANTIS study which also demonstrated that R_5_–R_20_ and AX were significantly correlated with asthma exacerbations in a milder asthma population. In addition, these authors developed an impulse oscillometry ordinal score, based on a composite of R_5_–R_20_, AX and X_5,_ that was directly related to the risk of exacerbations. A one-point increase in the ordinal score, indicative of increased small airways resistance, was associated with a 16% increased risk of exacerbations (34).

In our study, small airway dysfunction was also associated with poorer asthma control and increased symptom burden. This is consistent with the study by Abdo and colleagues that demonstrated the worst asthma control was observed in those with greatest increase in resistance and reactance absolute values measured by respiratory oscillometry (31). Similarly, the ATLANTIS study showed lower ACT scores were associated with higher AX and R_5_ and the impulse oscillometry ordinal score independently predicted the level of asthma control (27, 34).

These data support the proposal that small airways dysfunction should be considered as a treatable trait in asthma (35). To be considered a treatable trait, small airways dysfunction needs to be (A) clinically relevant, (B) identifiable and measurable and (C) treatable (36, 37). Our study adds to the body of evidence that satisfies the first two criteria in that it is identifiable and measurable using oscillometry and that small airways dysfunction as determined by the cutoffs applied. The associated increased risk of asthma exacerbations and poor asthma control further confirms its clinical relevance. The assessment of treatment response targeting small airways dysfunction is beyond the scope of our study. Nonetheless, raised resistance and reactance measured by oscillometry was found to be predictive of greater treatment response in a small Australian study of patients who received step-up treatment with ICS/LABA for poorly controlled asthma (17). With the availability of extra-fine particle size inhaled medications, we have the potential to treat small airways dysfunction as these inhalers provide good deposition of medication to the small airways (38, 39) which has been shown to be superior to that achieved by standard fine-particle size inhalers (40). Although the clinical superiority of extra-fine particle size inhaled medications have not yet been demonstrated in large prospective randomised controlled clinical trials targeting patient subgroup with small airways dysfunction, reassuringly the relevant trials in patients with asthma have not identified any downsides (demonstrating therapeutic non-inferiority) of using these extra-fine inhaled medications compared to standard particle size inhaled medications (41, 42). The potential to improve clinical outcomes with extra-fine particle size inhalers was reported in a meta-analysis of real-world observational studies where the use of extra-fine particle size ICS was associated with improved asthma control and lower risk of exacerbations than standard ICS inhalers (10). Similarly, a retrospective, observational study in patients with asthma treated with standard inhaler fluticasone propionate-salmeterol was compared to patients switched to extra-fine particle beclometasone dipropionate-formoterol found that both therapies were equally effective in preventing severe exacerbations, but those switched to the extra-fine particle therapy had improved odds of achieving overall asthma control and lower daily SABA usage and this was achieved with a lower daily ICS dosage (43). As such in our clinic, if small airway dysfunction is identified with IOS, consideration is given to trialing treatment with extra-fine particle size inhaled medications to determine if the patient experiences improvement in symptom control.

It is noteworthy that we are not proposing that respiratory oscillometry replace other lung function tests but should be used in conjunction with spirometry to gain a better understanding of pathophysiology to guide management (44). This is consistent with the findings of Chan and Lipworth that demonstrated that combining FEV_1_ with oscillometry measures indicative of small airways dysfunction, such as R_5_–R_20_ or AX, increases the power to identify patients at increased risk of both poor asthma control and frequent asthma exacerbations (24).

The main limitation associated with this study relates to the retrospective design and the fact that not all patients had relevant data for all parameters documented in their medical records. However, we applied a conservative approach, and when an outcome was not documented, this patient was excluded from that analysis rather than assuming that the outcome was favourable. In addition, at the time spirometry and oscillometry were performed in this study, assessments of normality were based on % predicted rather than *z*-scores. There are validated *z*-scores for some oscillometry parameters (21, 45), but not R_5_–R_20_, hence future research is required to determine what *z*-scores define abnormality for the most clinically relevant oscillometry parameters so that these correlations can be reassessed based on *z*-scores. It also needs to be acknowledged that oscillometry results are variable not only between IOS and FOT but also across different devices using the same technique (46, 47). This makes it difficult to draw conclusions across different studies for what defines abnormality and likely contributes to its slow uptake in clinical setting. Finally, a strength of this study is that it was conducted in a patient cohort referred to a tertiary severe asthma clinic. An associated weakness is these data may not be representative of patients with asthma managed in primary care. These data are transferable to patients referred to respiratory specialists who are best positioned to adopt impulse oscillometry into routine clinical practice with the aim of improving the management of this cohort of patients who generally have more severe or difficult to control asthma.

## Conclusion

Our study adds to the growing body of evidence that respiratory oscillometry is a sensitive measure of small airways dysfunction that can be routinely used in clinical practice to improve the assessment and management of patients with asthma referred to respiratory specialists. Small airways dysfunction is highly prevalent and correlates with the degree of airflow obstruction. In our study, frequency dependence of resistance (R_5_–R_20_) was sensitive in identifying small airways dysfunction and was most predictive of identifying patients at risk of moderate to severe asthma exacerbations and poor asthma control.

## Data Availability

The raw data supporting the conclusions of this article will be made available by the authors, without undue reservation.
